# ‘It's like a never‐ending diabetes youth camp’: Co‐designing a digital social network for young people with type 1 diabetes

**DOI:** 10.1111/hex.13690

**Published:** 2022-12-21

**Authors:** Kerstin Ramfelt, Boel Andersson Gäre, Ann‐Christine Andersson, Christina Petersson

**Affiliations:** ^1^ Jönköping Academy for Improvement of Health and Welfare, School of Health and Welfare Jönköping University Jönköping Sweden; ^2^ Qulturum—Center for Learning and Innovation in Healthcare Jönköping Sweden; ^3^ Futurum Jönköping Sweden; ^4^ Health Society and Health Science Malmö University Malmö Sweden

**Keywords:** adolescents, co‐design, improvement, social network, type 1‐diabetes, value creation

## Abstract

**Introduction:**

Living with a chronic condition such as type 1 diabetes (T1D) affects everyday life and support from others experiencing a similar situation can be helpful. A way to receive such support is to use an online network where people can connect and share experiences. Research has described the benefits of using such tools for connecting patients. The aim of this study was to describe the co‐design of a social network for young people with T1D and to describe their experiences when using this network.

**Methods:**

A co‐design approach was used, following three steps adapted from Sanders and Stappers (2008). In all, 36 adolescents with T1D participated. Data in the form of recordings and notes from telephone interviews, workshops and focus groups were collected and then analysed using content analysis. Numerical data from the digital platform were also used.

**Findings:**

For the interpersonal values, supporting, learning and relating to emerge, the framework of the network must be appealing and user‐friendly. The limits of time and place are eliminated, and there is a possibility for many more to join in.

**Conclusion:**

Co‐design ensures that what stakeholders think is important forms the basis for the design. The interpersonal values that are promoted are ones that only the exchange of lived knowledge and experience can generate. It is complementary to the support that healthcare professionals can offer; thus, this kind of social network is important for improved, coproduced care.

**Patient or Public Contribution:**

The participants in the present study were persons living with T1D. They were active co‐creators from the start to the end. An adult person with experience of living with T1D was involved as an advisor in the research team when drafting the manuscript.

## 1 INTRODUCTION

Type 1 diabetes (T1D) is a noncurable chronic condition that requires treatment and ongoing self‐management ‘24/7’. Adolescents with diabetes and their caregivers spend less than 1% of their time a year visiting a diabetes healthcare provider.^1^ Knowledge about the disease and the need for support are important factors in adolescents' lives; having a chronic condition may lead to the feeling of being different.^2^ Between the ages of 11 and 15, most young people perform much of the daily diabetes management, and in mid‐teens (ages 15–17) take responsibility almost entirely on their own.^3^ Living with T1D has a huge impact on adolescents' daily life,^4^ and they troubleshoot and make decisions in day‐to‐day activities on their own; therefore, they seek out and understand the importance of getting support from others in a similar situation.^5^, ^6^, ^7^ Even though healthcare professionals are aware of this need, it may be difficult to organize groups within the healthcare system and even if they do, few young people join in.^8^ Consequently, using other forms more focused on young persons, such as online communities, could represent one solution.

### 1.1 Online social networks

An online social community or network involves people sharing experiences and supporting each other in online activities.^9^, ^10^ Recent research describes the benefits of using different tools for connecting patients with chronic conditions to their peers.^11^ For example, young women with T1D found comfort in receiving social support on forums on the Internet from others in the same situation; it helped them maintain a balanced view of their lives and to manage life transitions.^12^ Adolescents and young adults with T1D who use social media in their everyday lives achieved better control compared to patients who did not use social media.^13^ In the United States, 23%–39% of young people seek peers online.^14^ Moreover, online social networks could be useful tools for patients and/or their caregivers to learn about blood glucose devices and receive technological assistance from other members. Through closed groups, members may help others in the network by spreading awareness about the condition itself, and providing emotional support and/or technical assistance when building on members' shared experiences.^15^

Existing research exploring diabetes online communities shows that people with T1D seek out diabetes online networks because it is challenging to identify a peer in real life. The shared experience has been mentioned as the most frequent topic discussed in several studies, and the sense of normality and validation of lived experiences are also central.^10^ The enthusiasm about using networks goes beyond information support and adds the value of emotional solidarity, shared feelings and experiences.^16^ The immediate response and orientations from other members give opportunity to acknowledge the community as a safe space. This is not possible in the contacts with healthcare providers.^17^ A recent scoping review of diabetes online communities reported promising results showing several benefits and relatively few negative outcomes. This points to the importance of participatory frameworks with the inclusion of users in the design and in the parameter‐setting stages, to better capture community elements and potentially increase social validity and usability of future networks.^10^ This indicates that the users should be involved in the design process when developing a social network. The aim of this study was to describe the co‐design process of a social network for young persons with T1D and to describe the experiences of using this network.

## 2 METHODS

### 2.1 Participants

A co‐design approach was used involving joint exploration and articulation of needs and solutions.^18^, ^19^ A sample of convenience^20^ was created, in which three outpatient children's departments, serving about 700 patients, in the southeast of Sweden were involved. The inclusion criteria were young persons between 13 and 17 years of age diagnosed with T1D at least 1 year before the initiative (Table 1). Twelve persons from each department were included (*n* = 36). Diabetes nurses at the participating departments provided contact information to parents whose adolescent children fulfilled the criteria. Members were informed that participation was voluntary, and that they could withdraw at any time without explanation, following the Declaration of Helsinki. Approval was given by the Swedish Ethical Review Authority (D.nr 2018/449‐32). Co‐designers were, in addition to the young persons, two researchers with experience of working with diabetes care, a software developer and two community managers from a health tech company.

**Table 1:** Description of participants' age and gender in the various steps during the study

Step	Activity	Invited	Participated	Age and background information	Gender
I	Telephone interviews	*N* = 36	36	13–17 years (median 15)	18 girls
				Range of years since diagnose 1–14 years (median 5)	18 boys
	Workshops	*N* = 36	21^a^	13–17 years	16 girls
					5 boys
III	Summarizing focus group interview or Telephone interviews	*N* = 36	6	13–17 years	5 girls
			3		1 boy
					3 girls

^a^ Reasons for not participating in the workshops were not having the time/opportunity on the day of the workshop or not interested in getting involved.

### 2.2 Procedure

**Figure 1:**
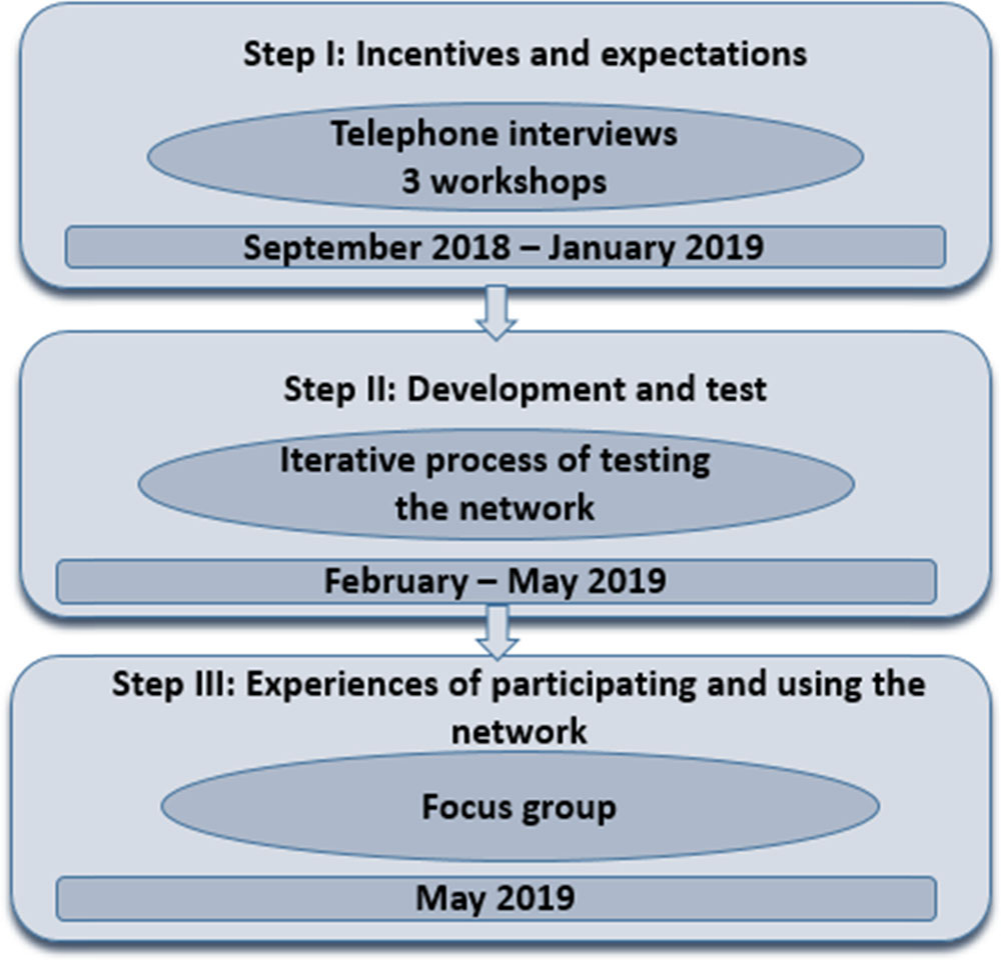
Description of the co‐design process adapted from Sanders and Stappers^19^ including a timeline for each step

The process of co‐design followed three steps, adapted from Sanders and Stappers^19^ (Figure 1). In the first step, telephone interviews were conducted to map out the general use of social media. These formed the basis for the semistructured workshops, where content and structure were enclosed for the online social network. The second step consisted of the iterative process of developing and testing the social network, and in the third step, a focus group was conducted to describe the experiences of using the network.

#### 2.2.1 Step I

Young persons with T1D were interviewed separately by phone (Table 1). The interviews were semistructured according to an interview guide (Table 2). Interviews were recorded and transcribed verbatim. The purpose was to understand these persons' use of social media and their ideas for developing a network for young persons with T1D. Then, they were invited to one of three semistructured workshops led by a software manager and two community managers.^21^ Of these, 21 persons (hereinafter referred to as members) participated in the workshops. The members had a meal together, while the software manager coordinated the discussion about habits of using social media in general: when, how, where and why they used different types of social media. The following discussion was then focused on what the intended social network would be like, including the content, technical features and user‐friendly functions. The discussion also focused on how the members wanted to use such a network and what type of content was important. Members wrote post‐it notes, which were grouped based on subject areas and clustered into different subjects, and were later used to create different areas within the social network. Two researchers observed the conversation and took notes.

**Table 2:** Interview guide used in the telephone interview in Step I

Question area	Questions
Background questions	Age, duration of diabetes diagnosis
	What kind of social media do you use today and how do you use them?
Thoughts about a social network for youths with T1D	If there was a social network for youths with T1D: what would be in it for you?
	What functions would you prefer?
	What would make you use such a network?
	Do you believe in the idea about such a network?
	Why?
	Why not?
	What are your thoughts about sharing your experiences of diabetes with others?
	What do you think about adults' presence in such a network?
Question about participation in the co‐designing process	Would you like to be involved in designing such a network together with other young people with diabetes and a software manager?

#### 2.2.2 Step II

A framework for social networks was used and was adjusted based on the members' views and requirements described in Step I. An invitation was sent to all 36 members to test the network over 12 weeks. During these weeks, an iterative process of developing content and structure and adjusting for errors was performed simultaneously. Members could create their own posts, write their opinions and views about the content and interact with each other. The Community Manager also posted questions, news and polls to engage members. All new posts led to a notification by email and a push notification in the app in members' smartphones. Members could comment on other persons' posts and they could ‘clap’, which had the same function as ‘like’ on other social media. There was also an ‘eye’ symbol with a number indicating how many members had read the post. Before the members got access to the community, each member was paired with another member as a ‘buddy’. Each buddy‐couple received tasks to discuss; the community manager initiated these tasks. This buddy function was a way to meet the need for individual support described in previous research.^22^ Data were collected from the community platform following the number of posts, comments and likes for members and the community manager.

#### 2.2.3 Step III

After the test period, all members were invited to a focus group interview led by the community managers. This was recorded and transcribed verbatim (Table 1). Two researchers observed and took notes during the focus group. The members were asked about their experiences, views and ideas for improvement of the network. The purpose of this focus group was to describe the members' experiences of participating in the development of the network and using the network.

#### 2.2.4 Data analysis

The telephone interviews in Step I were read several times to make sense of the data. Then, the text was coded by the first author and entered into a spreadsheet. The codes were grouped into subcategories by the first and last author. A thematic analysis was performed using an inductive approach strongly linked to the raw data.^23^ Numerical data (Step II) from the community platform were compiled. The focus group interview (Step III) was analysed using inductive content analysis.^24^ First, meanings and sentences were assigned a code. In the open coding phase, codes were grouped into categories and sorted under higher‐order headings. Similar subcategories formed higher‐order categories. To achieve trustworthiness, the other authors in the research group read and discussed the analysis.

## 3 RESULTS

In the telephone interviews (Step I), members described incentives for becoming a member of a social network, such as the opportunity to connect with others and share experiences together. Another incentive could be to provide and receive information and support. Furthermore, important prerequisites were described, especially the presence of an adult, that the network has many members and that all members have their own experience of T1D. The workshop's discussions focused on functions, opinions and the members' habits around using apps, the internet and smartphones. This formed the basis for designing the social network. Members stated the importance of simplicity, such as easy access and a clear overview. Quotes illustrating the participants' opinions are presented in Table 3.

**Table 3:** Quotes from telephone interviews and workshops illustrating the incentives and prerequisites for using a social network focused on young people with T1D (Step I)

Incentives and prerequisites	Quotes from interviews (Step I)
Connecting	I think it's a good idea, because then you can write with each other about the disease if you don't want to share it with someone else who might not understand it
T1D only	It feels good to know that it is only for those with diabetes. It might feel better then.
Support	I think it would be really, really nice, because since there aren't very many people with diabetes that you know, it would be great to have everyone gathered in one place because you sometimes feel quite alone
Share experiences	Maybe more that you post things there about tips and advice that you yourself think work
Presence of an adult	It might be more special if it's only for young people, but it's good if there are adults who can keep an eye in case something bad is shown

After clustering the subjects, five areas of different subjects emerged (news about diabetes, free questions, missions, polls and challenges—the last three created by the community manager). Then, members formulated rules for the social network. These included using a positive tone in the posts and avoiding negative reactions that could be interpreted as offensive. All members agreed that the community manager should be responsible for coordinating content, outsourcing assignments and polls and questions during the testing period.

All 36 members were invited to join the test period (Step II) and 33 logged in to the network. The members were encouraged to report any technical problems, which contributed towards improving the functionality simultaneously. Most posts and interactions were initiated by the community manager (87%). The posts concerning diabetes‐specific questions and information about diabetes received most feedback from the members. There was almost no contact between the buddie couples. Some of the members tried to get in touch but received no response from their buddy.

**Figure 2:**
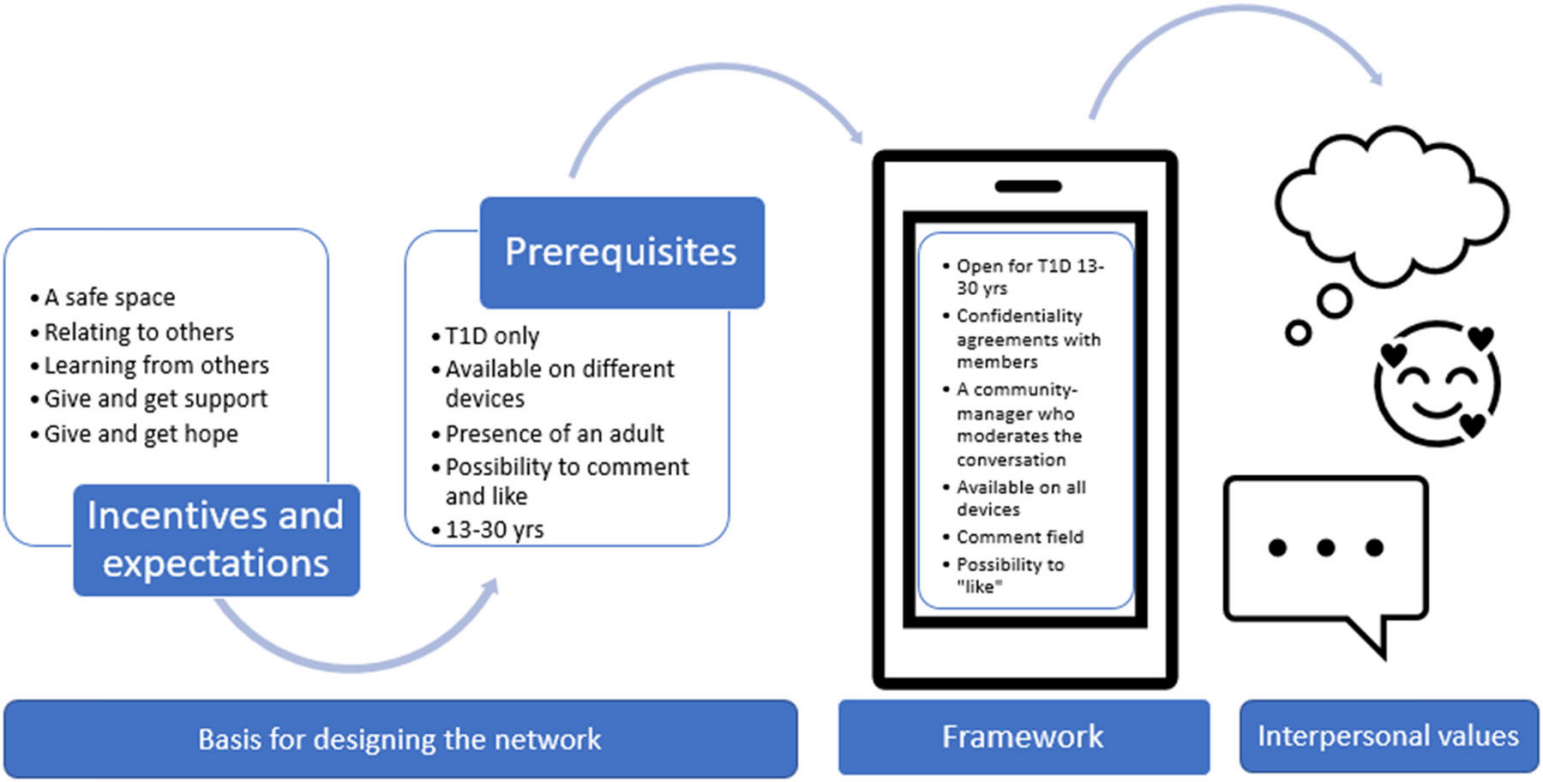
Results obtained from the three steps

To describe experiences of using the platform and of being a member of the network (Step III), all members were invited to a focus‐group discussion. The content analysis of this discussion revealed two categories: framework and possible interpersonal values (Figure 2 and Table 4). Quotes to illustrate the categories are presented in Table 4. The *framework* included ‘functionality’, exemplified by aspects such as accessibility and that the interface should be attractive irrespective of the devices used (smartphone, tablet or computer). Polls, posts and how notifications should be presented were discussed and also what kind of functionality was desirable. The members did not find any use for the buddy function, but it was argued that this kind of functionality may better suit members recently diagnosed with T1D.

**Table 4:** Quotes from the focus group that illustrate categories from the content analysis (Step III)

Category	Subcategory	Quotes from focus group (Step III)
Framework	Functionality	Great with that ‘eye’ showing how many people had read and reacted on the post
	Access and rules	I don't think we want to be there if there are a lot of parents just reading too
Interpersonal values	Relating	I have seen that there are more young people who feel a little like me. It's a bit what I take with me and it's probably what's nice that you have that app, that you have people who may be the same age as yourself as who you can easily reach
	Supporting	Yes, if I had had this [buddy function] when I got diabetes in the first place, maybe I had appreciated a buddy to ask—but you have that support anyway on the network
	Co‐learning	This thing about starting to drink [alcohol] … then it would have been good to have someone who was a bit older who had a bit more experience…

The second part about the framework was described as ‘access and rules’. The rules were about how to engage in the network and which members should be allowed. For example, members preferred that young adults were invited into the network. Issues about transition into adult care and how to prepare for moving away from parents could be important topics to raise in an extended network. The need for more activity from members was expressed since ongoing activity is important for a social network to serve its purpose. Another important issue was about not allowing parents to be part of the network, because their presence may deter members from engaging.

The second category included the interpersonal values that can be developed while on an online social network (see Table 4 and Figure 2). One of the interpersonal values described is about relating; that members share the same experiences and can understand each other in a deeper way. They described several examples in everyday life that those with their own experiences of living with diabetes could understand. It could be the feeling of having a bad day (such as a stubborn high blood sugar) and how others describe that feeling and relate to it. Another value was about supporting, that members could bolster each other. This was illustrated by the solidarity that they could offer each other when they feel a sense of hopelessness. This way of giving and receiving hope was expressed in terms of not being alone in the struggle with the condition in everyday life. Members pointed out that there is a lower risk of a judgemental attitude between those who share the same experience, which then added to the feeling of support. Finally, ‘co‐learning’ was mentioned, describing how members could learn different things from each other's lived experiences. This was more implicit, exemplified by the network being a platform for sharing concrete examples of how to do things, for example, what to consider when drinking alcohol and advice concerning food and physical activity. Members could learn from these concrete examples and apply them to their own situation.

Being a member on a social network has potentials for young persons with T1D. However, to accomplish those potentials described as interpersonal values in the present study, the network need an appropriate framework. When all these things come together, it could be considered as a safe space to meet others with the same experiences. It is not restricted to any physical place or an organized group of selected members. The limits of time and place are eliminated and there is a possibility for many more to join in. This was described by the members as ‘participating in a never‐ending diabetes youth camp’. Due to the nature of a virtual network, facilitated by a community manager, the safe space for meetup becomes a reality. A camp is limited to time and place and is therefore not available to everyone.

## 4 DISCUSSION

To our knowledge, this study is the first to use a co‐designed approach with young persons with T1D to create a digital network. Young people have a lot to contribute to the design as they can describe their incentives for using digital social media and how they use it.

The result shows that members want the presence of adults on the site as a prerequisite for the safe space that they value. This is in accordance with the idea of ‘facilitated networks’ as a possible configuration of value‐creating services.^25^ Teenagers can be empowered by sharing lived experiences with young adults with T1D,^26^ which was confirmed in our study. Members place a high level of trust in their peers and follow their advice about lifestyle changes,^11^ which indicates the importance of the presence of healthcare professionals in the network, to minimize the risk of incorrect advice being shared, which could otherwise be a risk.^13^ Members expressed the need for functionality, access and rules. Others have described that online networks need mentors, guides and moderators to be present. Such a presence can provide the necessary structure and direction to shift a negative or unproductive social networking process into one that could positively influence social support.^27^ In the current social network, the risk of bad behaviour and misinformation has been minimized by the presence of a community manager.

Continuous interactions within the network are needed, which are dependent on members' engagement and the willingness to share personal experiences.^27^ A critical minimum number of members has been confirmed by White et al.,^28^ who report that a rapid increase in membership and level of participation in the network indicates motivation and increases the possibilities for exchange of information. The low level of activity from and between members in this study may indicate that the test is too small or that the group was too homogeneous. Even if there is a small percentage of members who are active on a forum, it is worth keeping in mind that persons who choose not to post content or comment on others' postings may still benefit from observing or being part of the community.^29^

We found that membership in a social online network offers a platform to both seek out and provide tailored social support around diabetes management. Our results about the interpersonal values; supporting and co‐learning as incentives for being a member in the network is confirmed by previous studies.^16^, ^29^ Members highly value being part of a digital social network since it increases knowledge, improves self‐care and reciprocates emotional support.^10^, ^29^, ^30^ Knowledge about T1D can form the basis for successful self‐management.^27^ Belonging to a social network where members can receive emotional support and mutual reciprocity and being part of other members' lived experiences are important driving forces for using diabetes online social networks.^10^

To conclude, the findings in this study are consistent with previous literature about online networks for persons with T1D and provide a more in‐depth understanding of the nature of online social networks. The results can be applied to a wider perspective of online networks to foster peer‐to‐peer support for other chronic conditions as well. The use of co‐design adds the value of directing content and structure to meet the needs of potential new members in future networks. It also ensures that structure and content are designed based on what users consider important and not based on assumptions made by others.

### 4.1 Methodological considerations

A strength of this study was that we could work iteratively, due to the close collaboration between members, technology developers, community managers and researchers, although the several steps of data collection in the co‐design process were time‐consuming. Further, a potential limitation is that people who agree to participate may not be representative of the population. Members were recruited by the diabetes nurse, and we cannot control if the diabetes nurse may have asked specific persons to join the study due to special characteristics. This may have affected the sampling procedure. The members that were included in the present study had several years of experience of living with T1D, which may have influenced the design of the network. The needs can vary depending on how long a person has lived with diabetes. In addition, we started with a rather small group of members, which resulted in a low degree of activity in the network. On the other hand, this was part of the learning process and further cycles with a larger group of users are called for. The network was based on an existing technology framework, which may have affected the members' creativity in the design of features and appearance.

## 5 CONCLUSIONS

We identified the potential advantages of joining a network for adolescents with T1D. Relating, supporting and learning together is something that the exchange of lived knowledge and experience can generate. This cannot be provided by healthcare professionals. By using co‐design, it was possible to straightaway build on what the young persons described as important. The participation of a facilitating healthcare professional was considered necessary by the users, to make the network a safe space to share and learn from. For future research, we recommend exploring the content in the network that could provide information about what is important for a wider group of young persons living with diabetes, and to also use this as a channel for patient feedback to diabetes teams to enable them to make improvements towards better, coproduced care.

## CONFLICT OF INTERESTS

The authors declare that there are no conflict of interests.

## Data Availability

The focus group and interview data (transcripts) that support the study conclusions are unavailable for public access because informed consent to share the complete transcripts outside of the research team was not obtained from the study participants.
